# Implementation of intermittent preventive treatment in pregnancy with sulphadoxine/pyrimethamine (IPTp-SP) at a district health centre in rural Senegal

**DOI:** 10.1186/1475-2875-7-234

**Published:** 2008-11-07

**Authors:** Piero L Olliaro, Henriette Delenne, Moustafa Cisse, Malick Badiane, Alberto Olliaro, Michel Vaillant, Philippe Brasseur

**Affiliations:** 1 UNICEF/UNDP/WB/WHO Special Programme for Research and Training in Tropical Diseases (TDR), 20 avenue Appia, CH-1211 Geneva 27, Switzerland; 2 Dispensaire Saint Joseph, Mlomp, District d'Oussouye, Sénégal; 3 District Médical d'Oussouye, Sénégal; 4 UNIGE, Université de Genève, Switzerland; 5 Unité d'Epidémiologie Clinique et de Santé Publique, Centre d'Etudes en Santé, CRP-Santé, Luxembourg; 6 Institut de Recherche pour le Développement (IRD), Dakar, Sénégal

## Abstract

**Background:**

Intermittent preventive treatment with *s*ulphadoxine-pyrimethamine (SP) is recommended for reducing the risk of malaria in pregnancy and its consequences on mothers and babies (IPTp-SP). Indicators of implementation and effects of IPTp-SP were collected in a rural clinic in Southern Senegal.

**Methods:**

Women seen routinely at the antenatal clinic (ANC) of a rural dispensary during 2000–2007. Deployment of IPTp-SP started in January 2004. Inspection of antenatal and outpatient clinic registries of the corresponding period.

**Results:**

Between 1^st ^January 2000 and 30^th ^April 2007, 1,781 women of all gravitidities and parities attended the ANC with 965 deliveries (606 and 398 respectively since 1^st ^January 2004, when IPTp-SP was started.) 69% of women were seen ≥ 3 times; 95% received at least one dose and 70% two doses of SP (from 61% in 2004 to 86% in 2007). The first visit, first and second dose of SP occurred at a median week 20, 22 and 31. The probability of receiving two doses was > 80% with ≥ 3 antenatal visits and a first dose of SP by week 20.

The prevalence of maternal malaria was low and similar pre- (0.7%) and during IPTp (0.8%). Effects on of low birth weight (LBW, < 2.5 kg) were non-statistically significant. The prevalence of LBW was 10.8% pre- and 7.7% during IPTp deployment (29% risk reduction, p = 0.12).

Unfavourable pregnancy outcomes numbered 72 (7.5% of pregnancies with known outcome), including 30 abortions and 42 later deaths (late foetal deaths, stillbirth, peri-natal) of which 13 with one or more malformations (1.35% of all recorded deliveries).

**Conclusion:**

The implementation of IPTp-SP was high. Early attendance to ANC favours completion of IPTp-SP. The record keeping system in place is amenable to data extraction and linkage. A model was developed that predicts optimal compliance to two SP doses, and could be tested in other settings. Maternal malaria was infrequent and unaffected by IPTp-SP. The risk of LBW was lower during IPT implementation but the difference was non-significant and could have other explanations.

## Background

Acquiring malaria during pregnancy contributes to maternal illness and low birth weight (LBW, defined as birth weight below 2,500 grammes, caused by either prematurity or intra-uterine growth retardation), which constitutes the greatest risk factor for neonatal and infant mortality [1]. Intermittent Preventive Treatment with drugs given routinely to pregnant women irrespective of the presence of peripheral malaria parasites is part of the World Health Organization (WHO) strategy to reduce the risk of malaria in pregnancy and its consequences on the mother and her baby (IPTp) [2].

The concept of IPT is based on clearance of the existing (usually asymptomatic placental) infection and post-treatment prophylaxis, i.e. the suppression of new infections over a variable period of time, depending on the drug kinetics, blood levels and parasite susceptibility [3]. The only option currently available is sulphadoxine/pyrimethamine (SP, 1,500 mg/75 mg). Two doses of SP, one on the second trimester (after quickening) and one on the third trimester of pregnancy, are recommended by the WHO in areas of stable *Plasmodium falciparum *malaria transmission with low (< 10%) HIV prevalence.

Two recent systematic reviews showed that (a) chemoprophylaxis or IPT reduces antenatal parasite prevalence and placental malaria in women in all parity groups, and improves birth weight and possibly perinatal death in low-parity women [4]; (b) IPTp with SP remains effective even in areas of parasite resistance to antifolate drugs, though more doses may be required in the presence of HIV coinfection [5]. However, the efficacy of this intervention will likely be eroded by spreading parasite resistance to SP and alternative drugs should be sought [3,6].

While implementing this policy, the WHO recommends using outcome and impact indicators to document the rate of implementation in control programmes and effects of delivering IPTp on maternal and childhood health [2]. Relevant information was extracted from dispensary registries in the District of Oussouye, Casamance, Senegal, an area of moderate malaria transmission.

## Methods

Mlomp is a village of approximately 8,000 people in the District of Oussouye, Casamance, south-western Senegal. Malaria is meso-endemic in this area and increases during the rainy season (July to September). The entomological inoculation rate is 25 infected bites per person-year [7]. Malaria affects all ages, with a peak between 6–15 years of age [8]. Until 2000, clinically suspected malaria was treated with quinine or chloroquine; thereafter, a new policy of treating parasitologically confirmed malaria with a combination of artesunate and amodiaquine was gradually introduced [9]. Contrary to chloroquine, quinine, artesunate and amodiaquine [9,10], *s*ulphadoxine-pyrimethamine (SP) has not been used and no data on its efficacy and safety exist.

The village dispensary, which is run by the St Joseph's congregation, reports to the Oussouye medical district. The dispensary has an antenatal clinic (ANC) and a maternity clinic, in addition to its general dispensary activities. The ANC runs once a week. Pregnancies which cannot be adequately dealt with locally are referred to the district (Oussouye) or regional (Ziguinchor) hospital. A thin and thick smear is done every time a patient presents with signs/symptoms suggestive of malaria; for pregnant women, this would happen either at the ANC or, if outside ANC days, at the general clinic.

According to the policy in Senegal, every contact is recorded both on the general register of the dispensary and on the patient's health card. Each woman attending the ANC is recorded in the pregnancy register and is given an obstetric card, recording every visit and event. The occurrence of malaria is recorded on the general register and the women's health card. When she is next seen at the ANC, this event is also recorded on the pregnancy register and her obstetric card, and is also reported on a register of complicated pregnancies, along with other notable or untoward events. Deliveries are detailed on a delivery register, and the pregnancy outcome reported on the general pregnancy register and women's obstetric card.

The pregnancy, pregnancy complications and delivery registers of the dispensary covering the period 2000–2007 were inspected and information extracted and linked. Data extraction concerned all women with a first antenatal visit by 30^th ^April 2007 and the relevant pregnancy outcome up until 15^th ^August 2007.

Information on age, gravidity and parity of women, date and number of visits and SP administration, pregnancy outcome with date and birth weight was recorded. HIV status is detected systematically unless the woman refuses to consent and the result provided confidentially. If HIV positive, the woman is referred for treatment to the District hospital. Other routine examinations at the first visit are blood group, haemoglobin, Wasserman test for syphilis and albumin in the urine.

The one-way ANOVA was used to compare birth weights between years and number of IPTp doses. Continuous data were assessed for normality using the Kolmogorov-Smirnov test; if significant, data were log transformed and analysed with a student 't' test, if normally distributed, otherwise, with the Kruskal-Wallis test. Dichotomous variables were analysed using the chi-squared or Fisher's exact test (Freeman-Halton if more than two categories), if the expected counts were lower than five in any cell.

A logistic model was used to estimate the odds of receiving two against one SP dose. The baseline characteristics of the women in the study [year of the first antenatal visit (2004 as reference), age, gravidity and parity] were entered in the model. A sub-analysis, restricted to the subjects who had at least one SP dose was carried out with explanatory variables recorded during the current pregnancy (number of antenatal visits, week of first antenatal visit, week of the administration of the first and second SP dose). Non significant variables were eliminated based on the Wald test and the Likelihood Ratio test. Interactions between parameters in the residual model were tested. Points of equipoise were calculated from the logistic model formula based on the estimated coefficients of the parameters and their interactions.

All tests were two-tailed. A p-value < 0.05 was considered statistically significant. Data were recorded in Excel^® ^and analysed with the statistical package SAS^® ^System Version 9.1.3 (SAS Institute, Cary, NC, USA).

## Results

**Figure 1:**
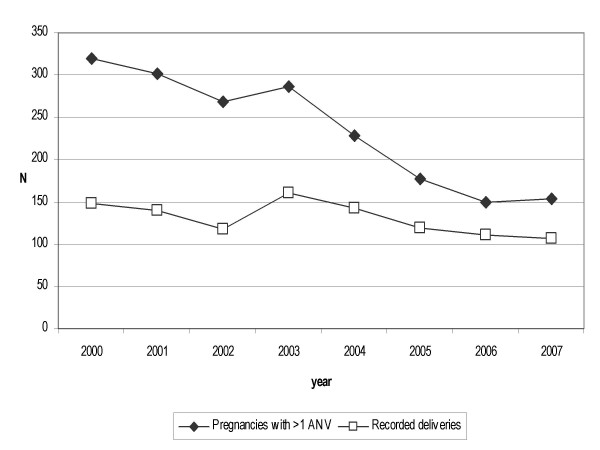
Pregnancies seen at the antenatal clinic of Mlomp and deliveries recorded during 2000–2007* (*projected). 2004 is the year of the beginning of the deployment of IPTp-SP.

Between 1^st ^January 2000 and 30^th ^April 2007, a total of 1,781 pregnant or suspected pregnant women made at least one visit at the ANC of St Joseph's dispensary in Mlomp, with 965 deliveries recorded (54%). The annual attendance decreased from 319 in 2000 to 150 in 2006 (projections for 2007 are stable with respect to 2006) (Figure 1). Effective January 2004, the policy of systematically offering women IPTp with sulphadoxine/pyrimethamine (SP) was implemented. The breakdown for the period 2000–2003 was 1,175 women seen at least once at the ANC and 567 deliveries (48% of the women seen at the ANC) vs. 606 women and 398 deliveries (66%) for 2004–2007 (chi-square p = 0.0001). Birth weight was available overall for 904 babies (94% of the recorded deliveries), 529 (93%) and 375 (94%) for 2000–03 and 2004–07, respectively.

**Figure 2:**
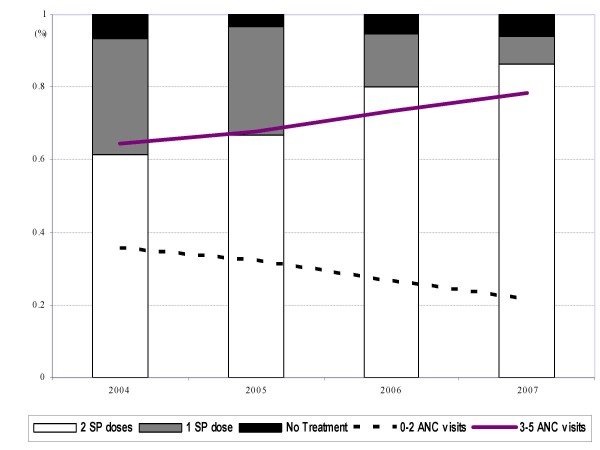
Rate of implementation of IPTp-SP and number of ANC visits in Mlomp between 2004–2007.

The 606 women attending the ANC from January 2004 had a median number of 3 (range 1–5) visits for each pregnancy; 417 (69%) were seen ≥ 3 times (from 65% in 2004 to 78% in 2007). Over the period 2004–2007, 574 (95%) received at least one dose of SP and 422 (70%) two doses (from 61% in 2004 to 86% in 2007). (Figure 2 and Table 1). The attendees were women with a median age of 28 years [range 15–48] who had previously had between 0 and 14 pregnancies.

**Table 1:** Performance indicators of implementation of IPTp in Mlomp

		**2004**	**2005**	**2006**	**Jan-Apr 2007**	**total**
women seen at ANC	N	228	177	150	51	606
at least 3 antenatal visits	N	147	120	110	40	417
	*%*	*64%*	*68%*	*73%*	*78%*	*69%*
at least 1 dose of SP	N	213	171	142	48	574
	*%*	*93%*	*97%*	*95%*	*94%*	*95%*
2 doses of SP	N	140	118	120	44	422
	*%*	*61%*	*67%*	*80%*	*86%*	*70%*
recorded deliveries	N	142	119	111	26	398
	*%*	*62%*	*67%*	*74%*	*51%*	*66%*

**Figure 3:**
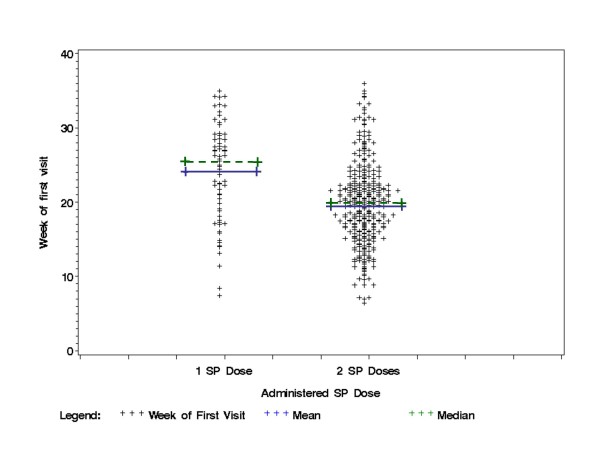
Distribution of weeks of first antenatal visit for the women who received one or two SP doses.

The median week of pregnancy at which the first ante-natal visit occurred was week 20 (quartiles 16–24, n = 394), and was week 22 (19–25, n = 392) for the first dose of SP and week 31 (27–33, n = 325) for the second dose. Women who had two SP doses had their first visit on a median week of 20.0 (quartiles 16.0–22.5, n = 325) compared to 25.6 (18.8–29.0, n = 67) for those who had only one dose (Figure 3). 221 (56%) women received SP according to the WHO recommended IPTp schedule of the first dose in the second trimester and the second dose in the third trimester.

The logistic model based on the baseline characteristics of the 567 women with at least one dose of SP showed that the year in which the first visit occurred was significantly associated with the probability of having two doses of SP (p < 0.0001) and was significantly higher in 2006 and 2007 than in 2004 (OR (95%CI) = 2.97 (1.70–5.18) for 2006 and 5.76 (1.97–16.88) for 2007). Age, gravidity and parity of the women had no influence.

When considering the 392 women with a recorded week of first visit and first SP dose, the probability of having a second dose of SP was significantly related to the number of antenatal visits, the week of the first visit and the week of first SP dose. The results of the logistic model are summarized in Table 2.

**Table 2:** Results of linear combinations estimation from the logistic model of two vs. one SP doses.

Contrast	Odds Ratio	95%CI		Pr > ChiSq
1 visit increase in the number of antenatal visits	51.72	8.21	325.80	< .0001
1 week increase in the week of first visit	2.25	1.50	3.38	< .0001
1 week increase in the week of first SP dose	0.96	0.73	1.25	0.7443
5 week decrease for the first antenatal visit in women who had 3 to 5 visits	31.92	5.15	197.80	0.0002
5 week decrease for the first antenatal visit in women who had 0 to 2 visits	57.83	7.61	439.50	< .0001
5 week increase for the first SP dose in women who had a first antenatal visit at week 30	10.79	4.74	24.56	< .0001
5 week increase for the first SP dose in women who had a first antenatal visit at week 10	2.57	1.10	6.02	0.0300

**Figure 4:**
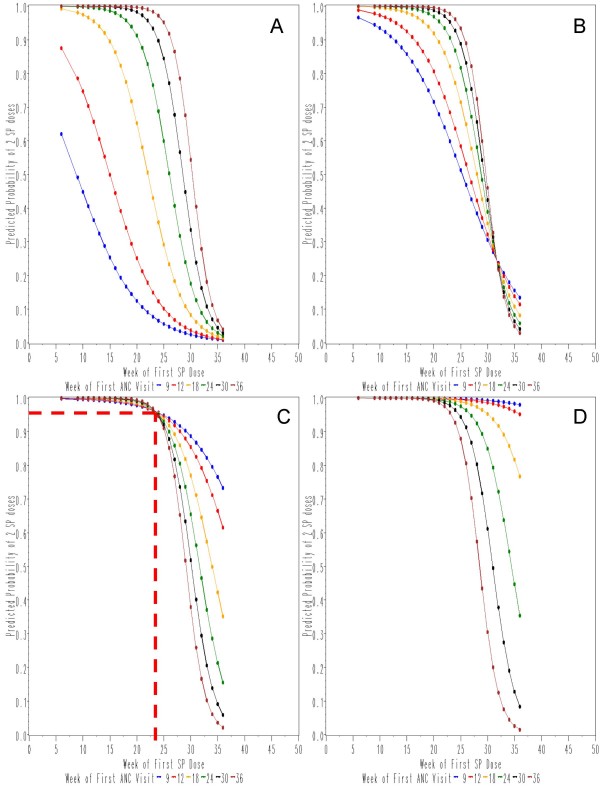
**Logistic model of the probability of receiving two SP doses for women with recorded delivery, date of first visit and SP administration.** The panels are for women with < 3 (A), 3 (B), 4 (C) and 5 (D) antenatal visits. For ease of reading only the calculated curves for weeks 9, 12, 18, 24, 30 and 36 of the first SP dose are presented. [Note: The point of equipoise is depicted as an example in panel C: when the number of antenatal visit is = 4, the probability of 2 SP doses is = 0.96 at the week of first SP dose = 23 when the week of first ANC is either 9, 12, 18, 24, 30 or 36].

The model does not estimate directly the Odds Ratio of the parameters that are included in the interactions and specific contrasts are estimated. Figure 4 depicts a simplified version of the model with selected weeks of first antenatal visit. For each and every week of first antenatal visit, the probability of having two SP doses increased with the number of visits. The model showed an interaction between the week of first visit and the number of visits, so that the occurrence of the first visit affected the probability of having two SP doses differently depending on how many visits took place thereafter.

Women with < 3 antenatal visits who had their first visit early had a low probability of a second SP dose. The probability of a second SP dose increased in this group if the first visit occurred later, but decreased for any week of the first antenatal visit, if the first SP dose was given late.

All women who underwent three antenatal visits had the same chance of a second SP dose if the first was given at 32 weeks (equipoise), whether the first antenatal visit was early or late. Before this week the relative effects of the week of first visit are similar to those described above for < 3 visits; beyond this week, the order is inversed and women who had their first visit early carried a higher chance of having a second SP dose than those who were seen later. The more the antenatal visits, the early the equipoise of probability distributions (23 in case of four visits, 15 if five). With the first SP dose on week 20 or before the probability of a second SP dose remained > 80% for any number of antenatal visits ≥ 3. (Figure 4)

Of the 606 women with a first antenatal visit between 1^st ^January 2004 and 30^th ^April 2007, the outcomes of 398 deliveries are recorded (66%); 170 women were lost to follow-up before delivery; in seven cases pregnancy was not confirmed or did not progress (possibly including early losses) and a record but no date exists for the remaining 33. No significant differences were observed for age, gravidity, parity, date of first visit or date of first SP dose between women with a recorded delivery and the others.

Overall, there were 28 unfavourable pregnancy outcomes (7% of the 398 known outcomes) including 14 spontaneous abortions (miscarriage occurring by month 4.5 of pregnancy), 12 stillbirths or late fetal deaths and two perinatal deaths (these 14 deaths represent 3.5% of the 398 pregnancies with recorded outcome). Six of these also had one or more obvious malformations (four anencephaly, one anophthalmia and clubfoot, and one with clubfoot = 1.5% of the 398 recorded pregnancy outcomes). Birth weight was available for 375 babies (median 3.1 kg, interquartile range 2.8–3.4 kg). In addition, two women were found to be HIV positive; they had uncomplicated pregnancies.

For comparison, 1,175 pregnancies and 567 deliveries (529 with birth weights) were recorded prior to starting IPTp during 2000–2003 with 44 unfavourable outcomes (7.8% of the 567 known outcomes), of which 16 were abortions, 21 stillbirths or late foetal deaths, and seven perinatal deaths (these 28 cases represent a 5% mortality for the 567 recorded pregnancy outcomes). Of these, seven children had one or more malformations (1.2%), namely two cases of hydrocephaly, one anencephaly (with clubfoot), two with clubfoot, one spina bifida and one case of respiratory tract malformation. The median birth weight during this period was 3.0 kg (quartile 2.8–3.4, n = 529). The observed difference in birth weight between 200–2003 and 2004–2007 was not significant (p = 0.43, Kruskall-Wallis)

Overall, 13 cases of parasitologically-confirmed maternal malaria were recorded between 2000–2007 among these women (one in 2000, four in 2001, one in 2002, two in 2003, one in 2004, one in 2005, two in 2006 and one in 2007). The incidence of malaria among women attending the ANC was 0.7% during 2000–03 (pre institution of IPTp) and 0.8% during the deployment of ITPp (Fisher 2-tailed test p value = 0.8). This refers to all cases of malaria in these women whether they occurred before their first antenatal visit or while being followed at the ANC.

Of the eight cases of malaria in pregnancy which occurred in women seen during 2000–2003, six had uncomplicated pregnancies and gave birth to normal babies (malaria diagnosed between months 3 and 7), one who had malaria in month 1 of pregnancy had a spontaneous abortion on month 3, and one had malaria in month 9 and delivered a stillborn baby five days after diagnosis and treatment. All were treated with parenteral quinine and resolved. Of the five cases occurring after 2004, malaria had occurred by month 4 of pregnancy and these women (aged 17–41) received subsequently two doses of SP. All were treated successfully and delivered healthy babies at term weighing 2.7–3.8 Kg.

**Figure 5:**
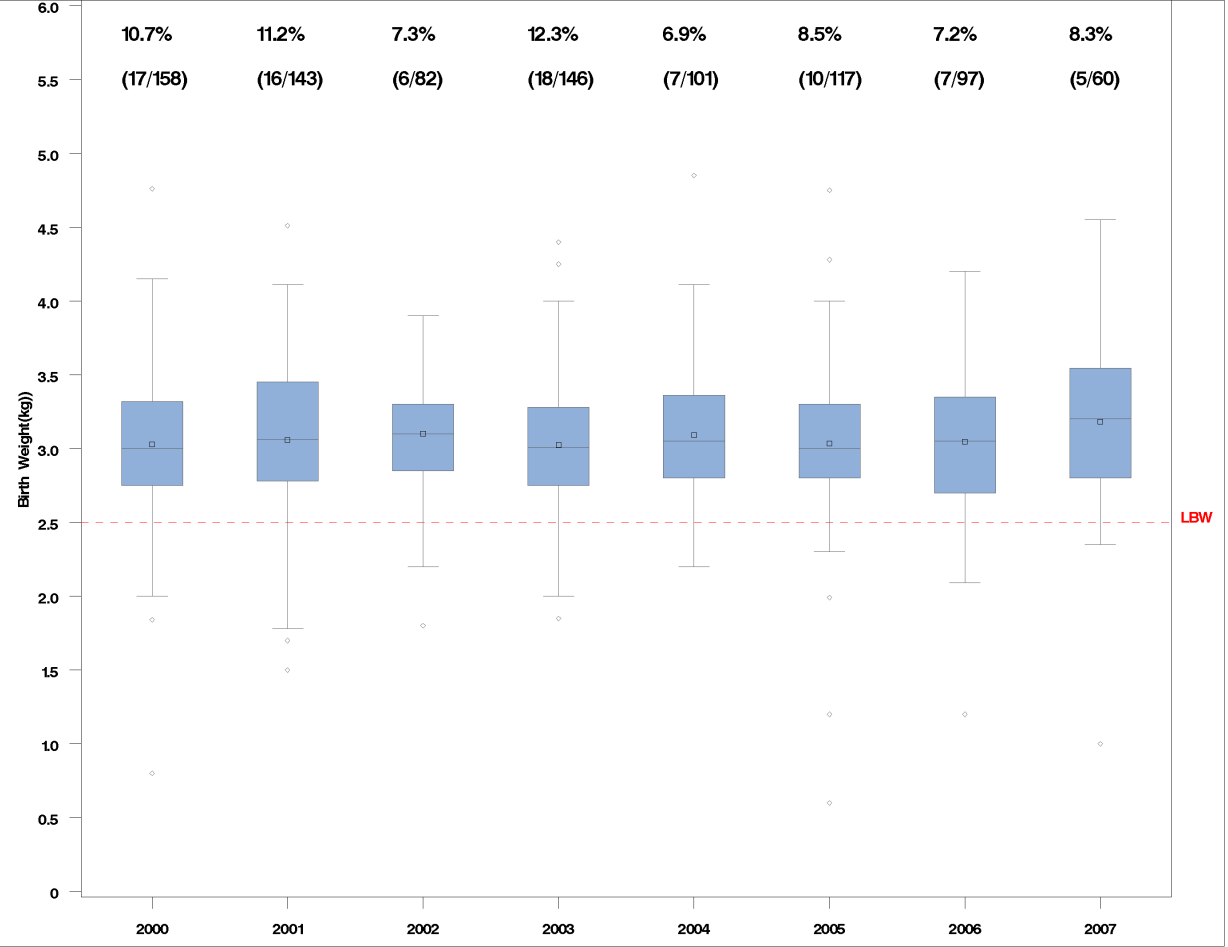
**Birth weight of neonates during 2000–2007.** Boxplots (mean, median, interquartile range) with whiskers (x standard deviation) and outliers. The dotted line represents defining a Low Birth Weight (LBW, birth weight < 2500 g). The % of newborn babies with LBW by year is displayed.

Low Birth Weight (LBW, defined as < 2.5 kg), was recorded in 86/904 (9.5%) of children born during 2000–2007, 29/375 (7.7%) of the neonates born to women seen in 2004–2007 and 57/529 (10.8%) in those born pre-2004 (Chi-square p-value = 0.12; OR [95%CI] = 0.69 [0.43–1.11]). The risk of LBW during 2004–07 was 29% lower than in 2000–03. Birth weights and percentage of LBWs by year are presented in Figure 5.

LBW was recorded in 22/309 (7.1%) of women who received two doses of SP, compared to 7/63 (11.1%) for one dose of SP, and 57/532 (10.7%) with no IPTp-SP (Chi-square p = 0.16) (Table 3). A logistic model showed no significant difference in the likelihood of LBW of being on IPTp-SP vs. no treatment (p = 0.16); the OR [95%CI] of LBW was 1.04 [0.45–2.40] with one SP dose and 0.61 [0.36–1.03] for 2 SP doses vs. no SP. The year or period (2000–2003 vs. 2004–2007) had no influence on the likelihood of LBW (p = 0.72 and 0.097, respectively; p = 0.13, OR [95%CI] = 1.44 [0.90–2.3]).

**Table 3:** Frequency of low birth weight (LBW) in newborns to women who received 2 doses, 1 dose of SP or no IPTp in Mlomp

		**Normal (=> 2.5 kg)**	**LBW (< 2.5 kg)**	**total**
2 SP doses (2004–07)	N	287	22	309
	%	92.9%	7.1%	
1 SP dose (2004–07)	N	56	7	63
	%	88.9%	11.1%	
no SP (2000–07)	N	475	57	532
	%	89.3%	10.7%	
All	N	818	86	904
	%	90.5%	9.5%	

Compared to no treatment, two doses of SP produced a 33.5% reduction in the prevalence of LBW (Absolute risk reduction: 3.59% [-0.30%, 7.48%]).

The prevalence of LBW among paucigravidae (≤ 2 pregnancies) was 11.7% compared to 8% in multigravidae (chi-square p = 0.4). Similarly, there was no difference in LBW in primigravidae. A residual logistic model (n = 375) showed that gravidity had a statistically non-significant protective effect on the probability of having a LBW baby (p = 0.38; OR [95%CI] = 0.93 [0.79–1.1]).

## Discussion

This article describes what can be achieved under current routine programmes in terms of both implementation of interventions and data collection during pregnancy. It shows that, with a good and motivated staff and compliant attendees, pregnant women can be routinely followed and IPTp can be effectively rolled out. The impact of IPTp on maternal malaria and birthweight is less clear in this setting.

Of the WHO recommended criteria to assess IPT in control programmes [2], implementation rate and birth weight could be measured at this rural health centre in Mlomp. In this area where malaria transmission is moderate, artemisinin-based combination therapy (ACT) is being deployed and HIV prevalence is low, the incidence of malaria and LBW were not significantly affected forty months into a high rate of implementation of IPTp-SP. It should be pointed out that the incidence of clinical malaria during pregnancy was very low both before and during IPTp-SP and that placental malaria was not assessed. However not significant and possibly influenced by other factors, LBW was 29% lower while rolling out IPTp-SP. It was also possible to collect information on pregnancy outcomes and obvious defects in the babies, in addition to maternal age, gravidity and parity.

The routine system in place in Senegal allows recording events occurring during pregnancy. Data redundancy is an inherent feature of this system (all or part of the information may be recorded in different registers and cards), which provides a degree of fault tolerance while making record linkage and data collation somewhat time-consuming.

The rate of implementation of IPTp-SP in Mlomp was high within few years of introduction of this practice; overall, 70% of the women attending the ANC received two doses of SP, increasing steadily from 61% on the first year of introduction to reach 86% within just 40 months of starting deployment. For reference, the initial target set by Roll Back Malaria (RBM) for 2005 was 60% of pregnant women at risk of contracting malaria to be given chemoprophylaxis or IPT [11]. In general, information on IPT coverage is limited. Hill & Kazembe [12] collated and analysed data available up to 2004. At that time, the implementation of IPTp was much lower than the RBM target in most countries. The coalition of Malaria in Pregnancy, East & Southern Africa, MIPESA) reported in 2002 coverage rates for one SP dose ranging from 33% in Uganda to 93% in Malawi and for two doses from 24% in Uganda to 68% in Zambia [13]. More recently, Crawley *et al *[14] report that now 34 of the 36 sub-Saharan African countries deemed eligible to IPTp deployment have adopted and 22 are implementing IPTp-SP. IPTp coverage varies and is difficult to quantify because delivery systems and data collection methodologies differ. In Uganda, new delivery approaches based on community mobilization and training resource persons achieved 67.5% compliance to two doses of SP, compared to 40% with the traditional approach [15].

Both Hill & Kazembe [12] and Crawley *et al *[14] also analyse the components and obstacles to the successful deployment of IPTp. Access to ANC services is one such element. In Mlomp, the median number of ANC visits was three (ranging from one to five) per pregnancy, and on average 69% of women were seen three times or more, figures which are very similar to those reported in the 1999 Demographic Health Survey (DHS) for the country (median 3.5 visits and 64% seen ≥ 3 times; for reference, the WHO recommends four antenatal visits [16]). Of note, the number of women with ≥ 3 visits in Mlomp increased steadily from 64% in 2004 to 78% in 2007, indicating improving performance. Overall, during the same period 34% had ≥ 4 visits. In the same review, these figures vary widely across Africa, from 2 to 4.8 visits and from 10 to 72% seen ≥ 4 times. Timing of attendance is also critical. In Mlomp, the median weeks women were pregnant at the first visit was 20 (i.e. in their 5^th ^month of pregnancy), which is later than the 1999 Senegal DHS data but within the range of the findings of the Hill & Kazembe review. Of the other critical elements identified by the review, here the turnout was high because antenatal services are in demand in this population and the customer is satisfied. The personnel at this Dispensary are highly motivated to include IPTp in the daily routine of ANC visits. The drug supply system is also good; there were no drug stock-outs so that the process can go on undisrupted.

According to WHO guidelines [2] and evidence from studies [5], programmes should aim for two doses of SP, while ongoing trials are further testing this schedule against monthly IPTp. The data from Mlomp indicate that, in general in this setting, the earlier the administration of the first dose of SP the higher the probability of receiving a second dose. However, this cannot be interpreted without taking into account the number of times a woman is seen during the course of her pregnancy; three antenatal visits appear to be critical.

Women who undergo only two visits and do so early have a low probability of a second SP dose because they do not comply with the ANC schedule and only come back for delivery. Those of them who do end up with two SP doses do so late into their pregnancy, which questions the real utility of the intervention in these cases. For women seen three times or more, the benefit of an early first SP dose on having a second dose is seen earlier in pregnancy as the number of visits increases. This also means that for these women, the earlier the first visit the more stable the probability of a second SP dose remains over time; 20 weeks appears to be the time of the first SP dose beyond which the chances of having two doses of SP start falling (and more rapidly so if the first visit is late). Of note, the median weeks of first ANC visit and first SP dose here were week 20 and 22, respectively, and 34% of these women had their first SP dose by week 20. Ideally, women should have their first ANC visit at nine weeks and have at least four visits in order to maximize the probability of two SP doses. With fewer than four ANC visits, the probability of two SP doses decreases rapidly (see Figure 4). The number of ANC visits is an important element to properly predict optimal conditions to receiving two doses of SP. Lastly, it should be noted that only 56% of women received one dose in the second and one in the third trimester as recommended by the WHO.

These data should be compared to other settings, and the model developed could be used to analyse and optimize service delivery at other sites. The effects of this intervention on mothers and babies are more difficult to assess. Mlomp is in an area of moderately intense malaria transmission where women in childbearing age are exposed to the risk of malaria – approximately one third of the malaria cases in Mlomp are 15–45 years old [8,9].

The deployment of IPTp-SP did not result in a reduction of maternal malaria, but cases were few both before and during the deployment of IPTp-SP. Placental malaria was not assessed. Over the study period malaria prevalence has declined in Mlomp. During 2000–2003, ca. 13,000 antimalarial treatments were administered per annum at the district health centre, compared to ca. 8,000 for 2004–2006. Similarly, the slide positive rate of patients presenting with fever declined from 44% to 28% (unpublished data extracted from the dispensary registers). Other concurrent interventions may have contributed: (i) the staggered implementation from 2000 of the artesunate+amodiaquine combination for parasitologically confirmed falciparum malaria [9] replacing quinine or chloroquine on presumptive diagnosis [8]; (ii) the distribution in the village of an average of 250 insecticide-treated bed nets (ITNs) per year since 2002, although there is no data on the use of ITNs. It is important to note that, even in this area of moderate transmission, maternal malaria tends to occur on or before month four of pregnancy, i.e. earlier than the first antenatal visit and the time at which IPTp is normally started here. This underlines the need for combined preventive measures including ITNs [17].

In this population, the prevalence of LBW in the absence of IPTp (years 2000–2003) was 10.8%, compared to 14.4% in the aggregate placebo arms of the studies included in ter Kuile et al systematic review [5]. Here, the prevalence of LBW dropped to 7.7% during IPTp deployment, irrespective of the number of SP doses (a 29% reduction) and was 7.1% for women who had two SP doses (vs. 10.7% if no IPTp, i.e. a 33.5% reduction). While statistically not significant, these results are clinically relevant, and are consistent with the 28.6% reduction overall found in the systematic review (36.9% when the analysis excluded the studies with concomitant use of ITNs). However, birth weight is multifactorial. There is indication of improved follow up of pregnancies in Mlomp. The proportion of pregnancies with three or more antenatal visits increased from 64% in 2004 to 78% in 2007; 46% of women seen at the ANC had a recorded pregnancy outcome in 2000 compared to 74% in 2006. In this setting, LWB was not significantly influenced by gravidity.

This paper provides also interesting data on pregnancy outcomes and defects. Obvious malformations in dead babies were seen in 1.35% of pregnancies with a recorded outcome (an incidence which is similar to that in developed countries [18]), with no difference between pre- and during IPTp. There was a preponderance of defects of the central nervous system and club foot; this could be due to the fact that these are more obvious defects to detect or could have occurred by chance (skewed distributions in limited series). Indeed proper training of health personnel to identify and describe malformations in a standardized way would be beneficial.

In conclusion, the implementation rate of IPTp-SP was very high in this rural clinic in south-western Senegal due to highly motivated personnel and good attendance and compliance by pregnant women. The record keeping system in place lends itself to data extraction and linkage. Optimal conditions for compliance to two SP doses could be modelled, and the model could be applied to other settings. The prevalence of LBW was lower by 29% during IPTp implementation years, but this effect was not statistically significant and could have other explanations. Effects on maternal malaria could not be demonstrated in this area of moderate malaria transmission and ACT deployment, but placental malaria was not studied. Policy makers should consider the merits and cost-effectiveness of IPTp deployment in areas of decreasing malaria transmission.

## Competing interests

The authors declare no conflict of interest. PO is a staff member of the WHO; the authors alone are responsible for the views expressed in this publication and they do not necessarily represent the decisions, policy or views of the WHO.

## Authors' contributions

All authors read and approved the final manuscript. PO contributed to the concept of the project; design of the protocol and analyses, reporting of the study, and prepared the manuscript. HD contributed personally to the treatment and follow-up of patients. MC and MB participated in the planning and supervised the implementation of the study. AO participated in designing the database and collected the data of the study. MV designed and conducted the analyses, contributed to the preparation of the manuscript. PB was the Principal Investigator of the study. He contributed to the concept, protocol, analysis and reporting of the study, and contributed to the preparation of the manuscript. He personally contributed to the treatment, follow-up of patients and quality control of the study.
